# Efficacy and tolerability of an undenatured type II collagen supplement in modulating knee osteoarthritis symptoms: a multicenter randomized, double-blind, placebo-controlled study

**DOI:** 10.1186/s12937-016-0130-8

**Published:** 2016-01-29

**Authors:** James P. Lugo, Zainulabedin M. Saiyed, Nancy E. Lane

**Affiliations:** 1 InterHealth Nutraceuticals, Benicia, CA USA; 2 Center for Musculoskeletal Health, University of California Davis Health System, 4625 2nd Avenue, Suite 2006, Sacramento, CA 95817 USA

**Keywords:** Knee function, Osteoarthritis, T regulatory cell, Undenatured type II collagen

## Abstract

**Background:**

Undenatured type II collagen (UC-II) is a nutritional supplement derived from chicken sternum cartilage. The purpose of this study was to evaluate the efficacy and tolerability of UC-II for knee osteoarthritis (OA) pain and associated symptoms compared to placebo and to glucosamine hydrochloride plus chondroitin sulfate (GC).

**Methods:**

One hundred ninety one volunteers were randomized into three groups receiving a daily dose of UC-II (40 mg), GC (1500 mg G & 1200 mg C), or placebo for a 180-day period. The primary endpoint was the change in total Western Ontario McMaster Universities Osteoarthritis Index (WOMAC) from baseline through day 180 for the UC-II group versus placebo and GC. Secondary endpoints included the Lequesne Functional Index (LFI), the Visual Analog Scale (VAS) for pain and the WOMAC subscales. Modified intent-to-treat analysis were performed for all endpoints using analysis of covariance and mixed model repeated measures, while incremental area under the curve was calculated by the intent-to-treat method.

**Results:**

At day 180, the UC-II group demonstrated a significant reduction in overall WOMAC score compared to placebo (*p* = 0.002) and GC (*p* = 0.04). Supplementation with UC-II also resulted in significant changes for all three WOMAC subscales: pain (*p* = 0.0003 vs. placebo; *p* = 0.016 vs. GC); stiffness (*p* = 0.004 vs. placebo; *p* = 0.044 vs. GC); physical function (*p* = 0.007 vs. placebo). Safety outcomes did not differ among the groups.

**Conclusion:**

UC-II improved knee joint symptoms in knee OA subjects and was well-tolerated. Additional studies that elucidate the mechanism for this supplement’s actions are warranted.

**Trial registration:**

CTRI/2013/05/003663; CTRI/2013/02/003348.

## Introduction

Osteoarthritis, which entails the destruction of joint cartilage and remodeling of the adjacent bone, is the most common form of arthritis affecting more than 25 million Americans [1]. Current therapies for OA include various over the counter analgesics, a number of nonsteroidal anti-inflammatory drugs (NSAIDs), intra-articular injections of corticosteroids or hyaluronic acid, plus tramadol and other opioid analgesics to relieve severe pain [2, 3]. While these therapies can alleviate symptoms in the near term, their ultimate impact on the pathophysiologic progression of OA is limited [4].

Previous studies reported UC-II to be efficacious for the treatment of arthritis [5, 6]. More recently, a statistically significant improvement in knee joint function over placebo was also reported in a clinical study comprising a group of healthy individuals, supplemented with UC-II, and who developed transient knee joint pain upon strenuous exercise [7]. These same individuals also took longer to experience pain after 120 days of supplementation. Based on these observations, the current study was designed to evaluate the efficacy of UC-II in knee OA subjects compared to placebo and to GC, which is a widely available supplement that is used for reducing joint pain.

## Materials and methods

### Investigational products

The study product UC-II® (Lot 1204004) was derived from chicken sternum. It was manufactured under current good manufacturing practice (cGMP) conditions using a patented process that preserved its native structure (Chick Cart Inc., Fort Smith, AR). Both glucosamine hydrochloride (GH) and chondroitin sulfate (CS) were purchased through Wilke Resources (Lenexa, KS). The Wellable group (Shishi City, Fujian) manufactured GH under cGMP and according to United States Pharmacopeia 26 specifications. Sioux Pharm (Sioux Center, IA) manufactured bovine-derived CS under cGMP. UC-II and GC were encapsulated in opaque, size “00” capsules with sufficient amounts of excipients (microcrystalline cellulose and silicon dioxide) such that they were sensory identical to placebo. InterHealth Nutraceuticals provided all study materials. All American Pharmaceutical (Billings, MT) verified the amount of active ingredients in the study capsules. Study materials were kept in a secure cabinet with access restricted to the site coordinator, the dispensing pharmacist, and the principal investigator.

### Study design

The objective of this randomized, double-blind, placebo-controlled clinical study was to evaluate the ability of UC-II to improve knee symptoms in OA subjects, as measured by overall WOMAC score, compared to placebo and to GC. The trial was conducted at 13 centers in southern India. Because of a limitation in synovial fluid sampling procedures at multiple clinical sites, the study was conducted under two separate study protocols. Study protocols were approved by each center’s Institutional Ethics Committee (IEC), and listed on the clinical trial registry of India as study protocols 003663 and 003348. Enrollment, randomization, and follow-up visits were identical for both protocols, and were carried out at days 1 (baseline), 7, 30, 60, 90, 120, 150 and 180 (Table 1). All investigators attended the same investigator meetings, used identical intake and data reporting forms, and were trained and monitored by the same group of clinical research associates.

**Table 1:** Protocol Schedule and Activities

Procedures common to both protocols	Screening (Visit 1)	Study period		
		Day 1 (Baseline Visit 2)	Days 7, 30, 60, 90, 120, 150 (Visits 3, 4, 5, 6, 7, 8)	Day 180 (Visit 9)
Signed Informed Consent	X			
Inclusion/Exclusion Reviewed	X	X	X	
Medical/Surgical/Medication History	X			
Physical Examination	X			
Vital Signs	X	X	X	X
Height^a^, Weight, BMI	X			X
Clinical Assessment for Knee Pain & Swelling	X	X	X	X
Knee Flexion Range of Motion		X	X	X
X-ray examination	X			
WOMAC Score	X	X	X	X
VAS Scale	X	X	X	X
LFI Score	X	X	X	X
Clinical Laboratory Tests (hematology, chemistry, urinalysis)	X			X
Urine Pregnancy Test (if applicable)	X		X	X
Serum biomarker analysis-COMP		X		X
Randomization Number Assigned		X		
Investigational Product Administration		X		
Dispense Subject Diary		X	X	
Collect/Review Subject Diary			X	X
Provide Directions for Concomitant Medication and Rescue Medication Use	X	X	X	
Dispense New Investigational Product		X	X	
Review Product Accountability			X	X
Assess use of Concomitant Medications		X	X	X
Adverse Events Assessed		X	X	X
Procedures Confined to Protocol 003348				
Synovial fluid biomarker—MMP-3 and IL-6		X		X
Serum biomarker analysis—CRP		X		X

^a^Height was measured only at Visit 1

Efficacy measurements were assessed at all visits and included WOMAC, VAS, and LFI indices. The knee flexion range of motion (ROM) test was performed at each visit. Subject diaries and study product were provided at all visits, except day 180 and were collected at all follow-up visits. Subjects were instructed to record daily the consumption of study product, use of rescue medication, as well as concomitant medications in the subject dairy for the entire duration of the study. Blood and urine were collected at screening and day 180. Pregnancy testing was done at screening and follow-up visits. Adverse events (AEs) were recorded using each subject’s diary inputs plus site visit questionnaires administered by intake personnel at all study visits.

### Clinical endpoints

The primary endpoint was defined as the change in total WOMAC score from baseline through day 180 for the UC-II group versus placebo and GC. Secondary clinical endpoints for both protocols were similar and included the change from baseline through day 180 versus placebo and GC for all endpoints including the following scores: (1) mean VAS; (2) mean WOMAC subscales; (3) LFI; and (4) knee flexion. Another endpoint included the change from baseline to day 180 for the serum biomarker cartilage oligomeric matrix protein (COMP). In protocol 003348, additional secondary endpoints included the change in serum biomarker, C-reactive protein (CRP) plus synovial fluid biomarkers interleukin (IL)-6, and matrix metalloproteinase (MMP)-3 from baseline to day 180.

### Study subjects

**Fig. 1:**
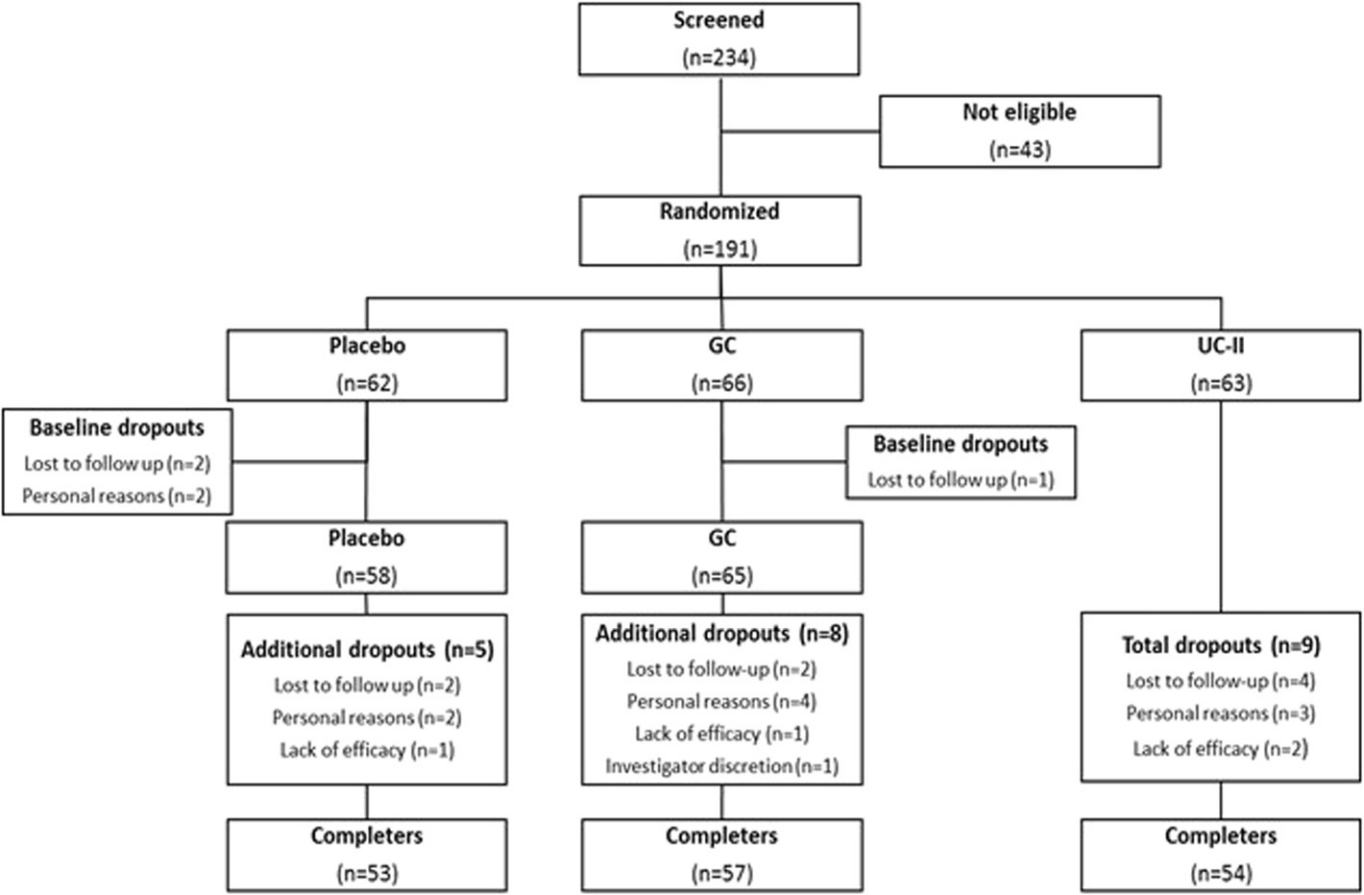
Enrollment and randomization flow chart

A total of 234 subjects were screened and 191 randomized (Fig. 1). Study inclusion criteria were 40–75 years-old male and female subjects, a body-mass index (BMI) of 18–30 kg/m^2^, moderate-to-severe OA by physical examination (crepitus, bony enlargements, joint swelling, etc.) in one or both knees, knee pain for at least 3 months prior to the start of the study, an LFI score between 6 and 10 and a VAS score of 40–70 mm 7 days after withdrawal from excluded medications, plus a knee radiograph that was graded as Kellgren and Lawrence (K-L) radiograph score of either 2 or 3 [8]. All OA diagnoses were confirmed by each study site investigator and noted in the subject’s case report form (CRF). In the case of bilateral knee involvement, the index knee used for the study was the one that presented with the most severe OA symptoms at baseline. Detailed inclusion–exclusion criteria are summarized in Table 2.

**Table 2:** Inclusion-exclusion criteria

Inclusion
• Ambulatory, 40–75 years of age, with a BMI of 18 to 30 kg/m^2^
• Females of childbearing age must agree to use a medically approved form of birth control and have a negative urine pregnancy test result throughout the study
• Female subjects of limited to no childbearing potential must be amenorrheic for at least 1 year or have had a hysterectomy, a bilateral oophorectomy, or both
• Unilateral or bilateral OA of the knee for greater than 3 months plus a Kellgren and Lawrence radiographic grade of 2 or 3
• VAS score during knee movement between 40–70 mm after 7 day withdrawal of excluded medications
• LFI score between 6–10 points after 7 day withdrawal of excluded medications
• Clinical laboratory results that are within normal range or considered not clinically significant by the Principal Investigator
• Be willing to participate in all scheduled visits, tests, and other trial procedures according to the clinical protocol
• Be willing to refrain from taking ibuprofen, aspirin or other NSAIDS, or any other pain reliever (OTC or prescription) during the entire trial other than acetaminophen (paracetamol) as rescue medication
• Provide a signed and dated informed consent indicating that the subject has been informed of all pertinent aspects and possible risks associated with participation in the trial
Exclusion
• History of hypersensitivity to the rescue medication or any of the products used in the study
• History of hypersensitivity to eggs, chicken or fowl, or shellfish
• History of inflammatory arthropathy, severe RA, OA (VAS score greater than 70), or Systemic Lupus Erythematosus
• Hyperuricemia (>440 μmol/L), past history of gout, or both
• Anticipation of surgery within the next 4 months
• Recent injury in the target knee (past 4 months)
• History of use for corticosteroid, indomethacin, glucosamine & chondroitin within 3 months of Visit 2; intra-articular treatments, including injections of corticosteroid or hyaluronic acid; consumption of Omega 3 fatty acids dietary supplements within 6 months preceding the treatment period (a 2-week washout period is allowed for subjects taking omega 3 fatty acid supplements)
• History of congestive heart failure
• Anticipated problems with product consumption
• Evidence or history of clinically significant hematological, renal, endocrine, pulmonary, gastrointestinal, cardiovascular, hepatic, neurologic diseases, or malignancies within the last 5 years
• High alcohol intake (>2 standard drinks per day) or use of recreational drugs (e.g., cocaine, methamphetamine, marijuana, etc.)
• Females who are pregnant or lactating or planning to become pregnant
• History of any mental illness that might impair the ability of subjects to provide a written informed consent
• Consumed acetaminophen (paracetamol), ibuprofen, aspirin or other NSAIDS, or any other pain reliever (OTC or prescription), or any natural health product, (excluding vitamins) within 7 days of first visit
• Participation in any clinical trials within 30 days prior to first visit

### Ethics, consent and permissions

Subjects were recruited after they reviewed, understood the study details, and then signed the IEC-approved consent form. The study conformed to the Declaration of Helsinki (version 1996).

### Randomization & blinding

Block randomization, consisting of nine individuals per block, was executed in a 1:1:1 ratio using random numbers generated by an independent statistician (SPSS version 16.0). Knowledge of the randomization code was limited to the statistician plus one QA monitor unrelated with the study. Each investigator was given opaque, sealed envelopes denoting single patient identity numbers, randomization codes, and supplementation regimen to be opened in case of an emergency. The code was broken after the clinical database was locked.

### Dosing regimen

Subjects ingested two blue pills in the morning with breakfast and two white capsules before bedtime. For the UC-II cohort, the two morning capsules were placebo, while the evening capsules contained 20 mg each of UC-II totaling 40 mg, which is identical to previously used clinical dose levels [5, 7]. This dose delivered 1.2 mg of undenatured type II collagen as determined by a newly developed and validated extraction-ELISA protocol (AIBiotech, Richmond, VA & Chondrex, Redmond, WA). For the GC group, the morning and evening doses delivered 750 mg of GH plus 600 mg of CS each totaling a daily dose of 1,500 mg of GH plus 1,200 mg of CS. The placebo group ingested identical numbers of blue and white capsules containing excipients only. Study bottles were labeled according to ICH-GCP and applicable local regulatory guidelines.

### Prior and concomitant therapies

Prior medications were documented at the screening visit by the study investigator. At each visit, study personnel reviewed subject diaries and questioned each participant on the use of any concomitant medications including those on the prohibited list. Prohibited medications included ibuprofen, aspirin, other NSAIDS, or any other pain relievers (OTC or prescription), plus any dietary supplements (excluding vitamins) that could support joint health. All concomitant medications used during the study was documented in the subject’s medical record by the study investigator then transcribed into their CRF by study personnel.

### Rescue medications

Acetaminophen was allowed at a dose of 500 mg twice daily. Participants were instructed to not take this medication within 48 h of an evaluation visit. Usage levels and timing was entered at each visit into the subject’s medical record by the study investigator. Study personnel transcribed this information into the subject’s CRF.

### Compliance and safety

Subjects were instructed to bring their bottles to each visit. Remaining capsules were counted and recorded in the subject’s CRF and accountability log. As a secondary measure of compliance, subjects completed a diary indicating daily dosing of the study products. Safety assessments were performed at all visits by the site investigator and staff (see Table 9).

### Study evaluations

WOMAC scores were determined using the WOMAC VA3.1 questionnaire containing 24 items grouped into three categories: pain, stiffness, and physical function (score range 0–2400). Each respective WOMAC subscale mean scores was determined by dividing the subscale score by the number of questions (5, pain; 2, stiffness; 17, physical function) it contained. The mean VAS score was determined using a VAS questionnaire containing 7 pain-related questions (score range 0–700), and then dividing the overall score by seven. LFI score was determined using an LFI questionnaire that assessed pain, walking distance, and activities of daily living, (score range 0–24). Knee flexion was measured using goniometry with the subject lying in the prone position and the leg to be tested positioned along the edge of the table [9].

### Synovial fluid biomarkers

Synovial fluid (~0.5 mL) was aspirated from the knee joint using an appropriate sized needle (18–24 gauge, depending on joint size). Harvested fluid was stored frozen until tested. IL-6 and MMP-3 levels were determined using the corresponding Duoset ELISA kits (R&D Systems, Minneapolis, MN).

### Serum biomarkers

COMP levels (Quantikine ELISA, R&D Systems) were determined in both study protocols. CRP levels (Latex COBAS INTEGRA, Roche Diagnostics GmbH, Mannheim) were assessed in protocol 003348. Serum was stored frozen until analyzed. Interassay and intrassay coefficients of variation for COMP and CRP were <5 %.

### Statistics

We verified, using 2-way analysis of variance (ANOVA), that the results of the two protocols could be combined into a single analysis by demonstrating there was no group by study interaction and that the magnitude of the efficacy observed under the two protocols was similar.

A modified intent-to-treat (mITT) analysis was used to assess the efficacy and safety outcomes that was defined *a priori*. This included all subjects who were randomized, consumed study product, and had at least one completed post-baseline visit. An analysis of covariance (ANCOVA), that included supplementation as a fixed factor and the corresponding baseline value of the variable being tested as a covariate, was used for assessing the overall statistical significance of the primary and secondary endpoints. Following ANCOVA, the Tukey-Kramer multiple comparison test was used for determining pairwise statistical significance and calculating 95 % confidence intervals. Also, a mixed model repeated measures (MMRM) analysis of the primary endpoint was performed to account for the multiple assessments obtained during this study. In addition, the method of trapezoids was used to calculate incremental area under the curve (iAUC) for all study groups. For iAUC estimation, missing values were imputed using the expectation-maximization algorithm in SAS. Rescue medication usage between groups was compared using logistic regression followed by pairwise comparisons using the Tukey-Kramer test. In addition, a stratified analysis of the primary endpoint was performed according to baseline serum COMP levels above and below the median value for this biomarker. Differences were considered significant if the resultant p-value was ≤0.05. An independent statistician performed the analyses and other calculations using SAS version 9.3 (Cary, NC).

## Results

### Demographics and baseline characteristics

Two hundred and thirty-four subjects were screened and 191 subjects who met the eligibility criteria were randomized to placebo (*n* = 62), GC (*n* = 66), or UC-II (*n* = 63) (Fig. 1). Per mITT criteria, 5 subjects were excluded from all analyses because they failed to present at any post-randomization visits resulting in an absence of clinical data. Table 3 summarizes the demographics of the remaining 186 subjects that were eligible for efficacy and safety analyses. Baseline subject characteristics, OA severity, serum CRP, COMP, IL-6 and other characteristics were similar among the three groups.

**Table 3:** Demographic and baseline characteristics of the trial subjects

Characteristics	Placebo (*n* = 58)	GC (*n* = 65)	UC-II (*n* = 63)
Sex ((n) male + (n) female)	28M + 30F	28M + 37F	33M + 30F
Age (years)	53.1 ± 1.02	52.6 ± 1.02	53.5 ± 0.99
Height (cm)	162 ± 1.00	161 ± 1.12	161 ± 0.89
Body weight (kg)	64.5 ± 1.20	66.0 ± 1.13	65.5 ± 1.12
Body mass index (kg/m^2^)	24.7 ± 0.40	25.5 ± 0.40	25.2 ± 0.37
Kellgren Lawrence radiographic score			
Grade 2 (n)	39	45	42
Grade 3 (n)	19	20	21
Lequesne's Functional Index	7.74 ± 0.12	8.02 ± 0.12	7.90 ± 0.13
Visual analog score (mm)	58.2 ± 0.97	59.1 ± 0.97	58.4 ± 0.99
Total WOMAC score	1382 ± 34.8	1396 ± 31.8	1398 ± 27.9
Mean WOMAC pain	56.9 ± 1.36	57.5 ± 1.33	58.1 ± 1.03
Mean WOMAC physical function	57.9 ± 1.51	58.5 ± 1.37	58.3 ± 1.24
Mean WOMAC stiffness	56.3 ± 1.63	57.3 ± 1.52	58.1 ± 1.32
Knee flexion ROM (°)	114 ± 1.62	114 ± 1.36	114 ± 1.57
Serum CRP (mg/L)^a^	5.29 ± 1.47	8.15 ± 1.79	3.35 ± 0.58
Serum COMP (ng/mL)^b^	325.2 ± 30.5	381.2 ± 44.1	334.6 ± 36.5
Synovial IL-6 (ng/mL)^c^	13.3 ± 4.73	13.9 ± 5.57	15.3 ± 6.04
Synovial MMP-3 (μg/mL)^d^	4.03 ± 1.20	2.54 ± 0.78	4.86 ± 1.74

Values presented as Mean ± SE

^a^Number of subjects used for analyses: 27, placebo; 29, GC; 29, UC-II

^b^Number of subjects used for analyses, 54, placebo; 58, GC; 55, UC-II

^c^Number of subjects used for analyses, 23, placebo; 24, GC; 21, UC-II

^d^Number of subjects used for analyses, 25, placebo; 27, GC; 23, UC-II

### Subject dropouts

One hundred and sixty four subjects completed the study: 53, placebo; 57, GC; and 54, UC-II. The 27 dropouts, which included the five subjects mentioned previously, were allocated across the three cohorts as follows: 9, placebo; 9, GC; and 9, UC-II. The final dropout rate was 14 %. Subjects’ dropout reasons are summarized in Fig. 1. No subject withdrew from the trial due to an adverse event attributable to any study product.

### Study product compliance

Compliance with daily dosing of study capsules exceeded 90 % for all cohorts (data not shown).

### Total WOMAC score

**Fig. 2:**
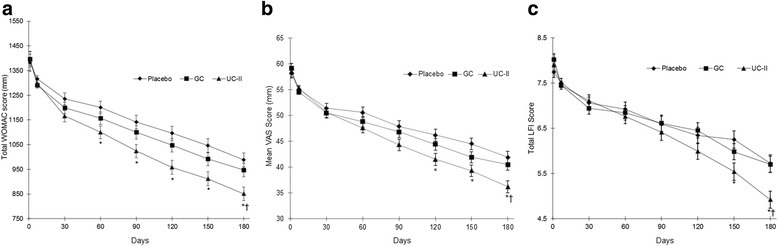
Total WOMAC score (**a**), Mean VAS (**b**), Total LFI (**c**) in the UC-II, GC and placebo groups over the 180-day study period. Values are presented as mean ± SE. *Significant difference between the UC-II (*n* = 54) and the placebo (*n* = 53) group, *p* < 0.05. †Significant difference between the UC-II (*n* = 54) and the GC group (*n* = 57), *p* < 0.05

The UC-II supplemented group had statistically significant changes in the total WOMAC score compared to placebo (−551 vs. −414; 95 % CI −232 to −42; *p* = 0.002) and GC (−551 vs. −454; 95 % CI −190 to −3; *p* = 0.04) at day 180 (Fig. 2a, Table 4). When the total WOMAC results were analyzed, using MMRM, to account for treatment by time interactions, there remained a statistically significant difference between the UC-II and the placebo groups (−514 vs. −397; 95 % CI −210 to −24; *p* = 0.0097; Table 4). An iAUC analysis also yielded statistically significant differences between the UC-II group versus placebo (−2042 vs. -1479; 95 % CI −1012 to −113; *p* = 0.0098; Table 4). No significant changes were observed between the GC and placebo cohorts regardless of the type of analytical model used.

**Table 4:** Change in total WOMAC score from baseline

Analytical method	Type of analysis	Time point (Days)	Placebo (*n* = 53)	GC (*n* = 57)	UC-II (*n* = 54)	*p* value (95 % CI)			
						Overall^a^	GC vs PBO	UC-II vs PBO^b^	UC-II vs GC
ANCOVA	mITT	180	−414 ± 28.5	−454 ± 27.5	−551 ± 28.2	0.002	0.56 (−134 to 53)	0.002 (−232 to −42)	0.04^c^ (−190 to −3)
MMRM	mITT	180	−397 ± 28.6	−452 ± 27.6	−514 ± 28.3	0.014	0.33 (−148 to 37)	0.0097 (−210 to −24)	0.25 (−153 to 30)
			(*n* = 58)	(*n* = 65)	(*n* = 63)				
iAUC	ITT	1 to 180	−1479 ± 137	−1751 ± 130	−2042 ± 132	0.014	0.33 (−718 to 174)	0.0098 (−1012 to −113)	0.26 (−727 to 146)

Values presented as Mean ± SE

*Abbreviations*: *PBO* placebo

^a^Overall p value was obtained by comparing the mean changes among the three groups using ANCOVA

^b^Significant difference between the UC-II and the placebo groups using Tukey-Kramer test

^c^Significant difference between the UC-II and the GC groups using Tukey-Kramer test

### Total WOMAC score based on baseline COMP levels

We found that subjects supplemented with UC-II, and presented with baseline COMP levels ≥285 ng/mL (median), had a greater reduction in the total WOMAC score than both placebo and GC groups with similar COMP levels under all modeling conditions (Table 5). For study participants with baseline COMP levels <285 ng/mL, no significant differences between the study groups were noted. Interestingly, we did observe a smaller placebo effect among individuals with baseline COMP levels ≥285 ng/mL as compared to those with <285 ng/mL (28 % vs 32 %). Despite this, UC-II efficacy, as defined by a reduction in overall WOMAC score, was higher in subjects with COMP levels ≥285 ng/mL versus subjects with COMP levels < 285 ng/mL (43 % vs 36 %).

**Table 5:** Stratified analysis for change in total WOMAC score based on baseline COMP levels

COMP (ng/mL)	Analytical method	Type of analysis	Time point (Days)	Placebo (*n* = 27)	GC (*n* = 28)	UC-II (*n* = 27)	*p* value (95 % CI)			
							Overall^a^	GC vs PBO	UC-II vs PBO	UC-II vs GC
≥285	ANCOVA	mITT	180	−368 ± 41.7	−396 ± 40.9	−574 ± 41.6	0.002	0.88 (−168 to 112)	0.002^b^ (−347 to −65)	0.009^c^ (−317 to −38)
	MMRM	mITT	180	−351 ± 44.1	−398 ± 41.1	−540 ± 44.2	0.006	0.71 (−188 to 94)	0.006^b^ (−330 to −48)	0.048^c^ (−282 to −1)
	iAUC^d^	ITT	1 to 180	−1351 ± 212	−1582 ± 204	−2384 ± 207	0.003	0.72 (−934 to 473)	0.002^b^ (−1741 to −325)	0.02^c^ (−1498 to −107)
				(*n* = 26)	(*n* = 29)	(*n* = 26)				
<285	ANCOVA	mITT	180	−463 ± 38.8	−508 ± 36.6	−526 ± 38.7	0.48	0.67 (−173 to 82)	0.49 (−195 to 68)	0.94 (−145 to 109)
	MMRM	mITT	180	−442 ± 38.2	−493 ± 37.3	−521 ± 38.1	0.34	0.60 (−178 to 76)	0.32 (−208 to 50)	0.86 (−155 to 100)
	iAUC^e^	ITT	1 to 180	−1626 ± 185	−1908 ± 178	−1902 ± 185	0.49	0.52 (−896 to 333)	0.55 (−902 to 350)	0.99 (−607 to 618)

Values presented as Mean ± SE

^a^Overall p value was obtained by comparing the mean changes among the three groups using ANCOVA

^b^Significant difference between the UC-II and the placebo groups using Tukey-Kramer test

^c^Significant difference between the UC-II and the GC groups using Tukey-Kramer test

^d^Number of subjects used for analyses, 27, placebo; 29, GC; 28, UC-II

^e^Number of subjects used for analyses, 27, placebo; 29, GC; 27, UC-II

### WOMAC mean subscores—pain, stiffness and physical function

At day 180, we observed significant reductions in all three WOMAC subscales for UC-II group compared to placebo (Table 6): pain (24.0 vs. 17.0; 95 % CI −11.1 to −2.8; *p* = 0.0003), stiffness (23.8 vs. 17.8; 95 % CI −10.4 to −1.6; *p* = 0.004), and physical function (22.5 vs. 17.3; 95 % CI −9.3 to −1.3; *p* = 0.007). The UC-II cohort also had significant reductions in WOMAC pain (24.0 vs. 19.2; 95 % CI −8.9 to −0.7; *p* = 0.016) and stiffness (23.8 vs. 19.4; 95 % CI −8.7 to −0.1; *p* = 0.044) at day 180 compared to GC.

**Table 6:** Reduction in mean WOMAC subscores in placebo, GC and UC-II groups over 180 days

Parameter reduction	Day	Placebo (*n* = 53)	GC (*n* = 57)	UC-II (*n* = 54)	*p* value			
					Overall^a^	GC vs PBO	UC-II vs PBO^b^	UC-II vs GC^c^
WOMAC pain	7	3.21 ± 0.58	4.57 ± 0.54	3.88 ± 0.55	-	-	-	-
	30	6.61 ± 1.04	7.89 ± 1.00	9.18 ± 1.01	-	-	-	-
	60	8.17 ± 1.10	10.1 ± 1.07	12.7 ± 1.09	0.0149	-	0.011	-
	90	11.2 ± 1.17	12.7 ± 1.14	16.4 ± 1.16	0.0063	-	0.0059	-
	120	12.9 ± 1.28	15.6 ± 1.22	19.9 ± 1.26	0.0005	-	0.0004	0.040
	150	15.0 ± 1.21	17.5 ± 1.16	21.5 ± 1.20	0.0007	-	0.0006	0.047
	180	17.0 ± 1.25	19.2 ± 1.20	24.0 ± 1.23	0.0003	-	0.0003	0.016
WOMAC stiffness	7	3.47 ± 0.64	4.22 ± 0.61	4.24 ± 0.62	-	-	-	-
	30	6.81 ± 1.10	8.76 ± 1.05	9.28 ± 1.07	-	-	-	-
	60	9.36 ± 1.28	11.5 ± 1.25	13.1 ± 1.27	-	-	-	-
	90	11.3 ± 1.36	13.8 ± 1.32	17.0 ± 1.35	0.0158	-	0.010	-
	120	13.6 ± 1.40	15.0 ± 1.34	20.0 ± 1.39	0.0035	-	0.0039	0.029
	150	15.5 ± 1.32	17.7 ± 1.26	21.3 ± 1.31	0.0079	-	0.0058	-
	180	17.8 ± 1.31	19.4 ± 1.27	23.8 ± 1.30	0.0043	-	0.004	0.044
WOMAC physical function	7	3.17 ± 0.56	4.14 ± 0.53	3.91 ± 0.53	-	-	-	-
	30	6.30 ± 1.00	7.80 ± 0.96	9.26 ± 0.98	-	-	-	-
	60	7.75 ± 1.08	9.50 ± 1.05	11.9 ± 1.07	0.0278	-	0.020	-
	90	10.4 ± 1.17	12.1 ± 1.14	15.1 ± 1.16	0.0182	-	0.0136	-
	120	12.7 ± 1.20	14.5 ± 1.15	17.9 ± 1.19	0.0083	-	0.0064	-
	150	14.8 ± 1.19	16.9 ± 1.14	20.0 ± 1.18	0.0078	-	0.006	-
	180	17.3 ± 1.21	18.8 ± 1.16	22.5 ± 1.20	0.0068	-	0.007	-

Values presented as Mean ± SE

^a^Overall p value was obtained by comparing the mean changes among the three groups using ANCOVA

^b^Significant difference between the UC-II and the placebo groups using Tukey-Kramer test

^c^Significant difference between the UC-II and the GC groups using Tukey-Kramer test. ‘-’denotes a non-significant statistical outcome

### Mean VAS score

The UC-II supplemented group had a significant decrease in mean VAS score at day 180 (Fig. 2b) versus both placebo (22.6 vs. 17.0; 95 % CI −9.5 to −1.8; *p* = 0.002) and GC (22.6 vs. 18.4; 95 % CI −8.0 to −0.4; *p* = 0.025). In contrast, the GC group was not significant compared to placebo at any time.

### LFI score

A significant reduction was observed in the LFI score for the UC-II group at day 180 versus placebo (2.9 vs. 2.1; 95 % CI −1.4 to −0.2; *p* = 0.009; Fig. 2c). UC-II supplementation also has a greater improvement in LFI score versus GC (2.9 vs. 2.2; 95 % CI −1.4 to −0.2; *p* = 0.008). No significant change was observed between the GC and placebo cohorts. Improvement in the total LFI score for the UC-II group was attributed to a significant reduction in the LFI subscale for daily activities at day 180 (*p* = 0.004 vs. placebo; *p* = 0.013 vs. GC, data not shown).

### Knee flexion

No significant differences were observed between the study groups (data not shown).

### Serum biomarkers

A significant increase in the final CRP levels versus baseline occurred in all three cohorts (*p* = 0.001). However, no statistical difference between the three cohorts (Table 7; *p* > 0.05) was noted. The scientific reason behind this increase is not well understood. A significant decrease in serum COMP levels was seen in all groups versus baseline (*p* = 0.04) with no significant changes between groups.

**Table 7:** Change from baseline to day 180 in serum and synovial fluid biomarkers

Matrix	Parameter reduction	Day	Placebo (n)	GC (n)	UC-II (n)
Serum	COMP (ng/mL)	180	−51.2 ± 31.3 (53)	−56.5 ± 36.0 (56)	−69.6 ± 40.8 (53)
	CRP (mg/L)	180	15.1 ± 6.33 (26)	9.09 ± 5.36 (28)	13.0 ± 4.64 (28)
Synovial	IL-6 (ng/mL)	180	−9.54 ± 4.83 (23)	−9.72 ± 5.28 (24)	−11.8 ± 5.37 (21)
	MMP-3 (μg/mL)	180	−2.24 ± 1.26 (25)	−0.93 ± 0.79 (27)	−2.67 ± 1.85 (23)

Values presented as Mean ± SE. Statistical analysis was performed on log transformed and baseline adjusted values. No significant differences were observed between the study groups (*p* > 0.05)

### Synovial fluid biomarkers

Similar non-significant decreases in IL-6 and MMP-3 levels were noted for all cohorts (Table 7).

### Rescue medication usage

The number of subjects that used rescue medication was significantly lower in the UC-II group compared to placebo (Table 8; *p* = 0.001). Sixty individuals used rescue medications, at least once, during the study. Twenty-eight of these users were from the placebo group, 21 and 11 were from the GC and UC-II cohorts, respectively.

**Table 8:** Number of subjects reporting use of rescue medication

Day	Placebo	GC	UC-II
7	11/58	12/65	3/63
30	18/58	7/63	4/61
60	12/58	9/61	6/59
90	12/56	8/59	3/57
120	13/54	13/59	7/55
150	10/54	12/59	3/55
180	11/53	7/57	4/54
Entire study period	28/58	21/65	11/63^a^

The table summarizes the number of unique individuals reporting the use of rescue medication. Data presented as number of subjects using rescue medication / total number of subjects observed. ^a^statistically significant versus the placebo (*p* = 0.001) based on pairwise Tukey-Kramer multiple comparison test. The overall group effect p-value was 0.002 using logistic regression

### Safety assessments

No clinical or statistically significant changes were reported for any of the hematologic, blood biochemistry or vital signs results (Table 9). No significant changes were noted for the urinalyses results (data not shown).

**Table 9:** Safety parameter assessment at baseline and day 180 in placebo, GC and UC-II groups

Parameter (Units)		Baseline						Day 180					
	Normal range	Placebo (*n* = 58)	GC (*n* = 65)	UC-II (*n* = 63)	*p* value GC vs PBO	*p* value UC-II vs PBO	*p* value UC-II vs GC	Placebo (*n* = 53)	GC (*n* = 56)	UC-II (*n* = 53)	*p* value GC vs PBO	*p* value UC-II vs PBO	*p* value UC-II vs GC
Hematology													
Hemoglobin (gm/dL)	12.1–17.2	12.1 ± 0.22	11.9 ± 0.21	12.1 ± 0.20	0.7613	0.9948	0.8095	12.7 ± 0.24	12.4 ± 0.20	12.7 ± 0.18	0.4454	0.9727	0.5851
ESR (mm/h)	0–29	21.1 ± 1.77	23.9 ± 2.18	17.5 ± 1.56	0.7629	0.1034	0.0144	15.1 ± 1.24	17.0 ± 1.91	13.6 ± 1.28	0.9424	0.5364	0.3387
RBC (million/mm^3^)	4.7–6.1	4.29 ± 0.08	4.21 ± 0.08	4.33 ± 0.09	0.7747	0.9388	0.5498	4.32 ± 0.08	4.25 ± 0.08	4.37 ± 0.08	0.7935	0.8946	0.5129
WBC (/mm^3^)	4500-10,000	7979 ± 234	8248 ± 222	7795 ± 249	0.7020	0.8483	0.3523	7984 ± 204	7981 ± 209	7769 ± 204	1.0000	0.7706	0.7639
Platelet count (x100000/mm^3^)	1.5-4.5	2.77 ± 0.08	2.84 ± 0.08	2.78 ± 0.08	0.7837	0.9946	0.8319	2.77 ± 0.07	2.84 ± 0.07	2.77 ± 0.09	0.8304	0.9993	0.8113
Liver Function													
Albumin (gm/dL)	3.5–5.5	3.98 ± 0.06	4.02 ± 0.06	3.94 ± 0.06	0.8957	0.9089	0.6503	4.00 ± 0.05	4.03 ± 0.05	3.96 ± 0.04	0.8931	0.8902	0.6292
ALP (IU/L)	44–147	117 ± 5.74	118 ± 5.84	115 ± 5.57	0.9871	0.9838	0.9404	123 ± 5.72	116 ± 5.49	115 ± 5.59	0.5622	0.4847	0.9890
SGOT (U/L)	10–34	25.2 ± 0.93	24.0 ± 0.97	24.4 ± 0.60	0.5778	0.7796	0.9421	24.6 ± 0.73	23.9 ± 0.81	23.9 ± 0.65	0.7711	0.7930	0.9995
SGPT (U/L)	5–35	25.9 ± 1.23	25.0 ± 1.40	24.1 ± 0.95	0.5977	0.6004	1.0000	24.5 ± 0.94	24.3 ± 1.00	23.3 ± 0.99	0.9688	0.7119	0.8427
Total bilirubin (mg/dL)	0.3–1.9	0.78 ± 0.08	0.69 ± 0.03	0.72 ± 0.03	0.5376	0.9424	0.7343	0.72 ± 0.03	0.67 ± 0.03	0.78 ± 0.04	0.4243	0.6098	0.0718
Cardiac Function													
Systolic BP (mm Hg)	<120	125 ± 1.28	127 ± 1.35	127 ± 1.21	0.5980	0.7320	0.9752	127 ± 1.18	125 ± 1.33	128 ± 1.22	0.7263	0.8949	0.4409
Diastolic BP (mm Hg)	< 80	81.2 ± 1.19	80.2 ± 0.83	81.7 ± 1.02	0.7544	0.9283	0.5094	80.2 ± 1.03	80.5 ± 1.07	78.9 ± 0.96	0.9877	0.6233	0.5180
Pulse rate (beats/min)	60–100	80.0 ± 0.92	79.6 ± 0.98	80.3 ± 0.99	0.9149	0.9719	0.7956	80.0 ± 0.89	78.2 ± 0.82	79.2 ± 1.03	0.3201	0.8018	0.6989
Renal Function													
Creatinine (mg/dL)	0.6–1.3	1.00 ± 0.03	1.01 ± 0.04	0.96 ± 0.03	0.9995	0.5767	0.5778	0.96 ± 0.03	0.95 ± 0.02	0.96 ± 0.02	0.9904	0.9846	0.9508
BUN (mg/dL)	6–24	18.1 ± 1.08	18.0 ± 1.11	18.0 ± 1.15	0.9929	0.9878	0.9992	18.6 ± 1.11	17.8 ± 1.09	17.9 ± 1.02	0.7602	0.7953	0.9985

Results are presented as Mean ± SE. Normal ranges were obtained from Medline^a^ and the Mayo Clinic^b^. Data was analyzed using ANCOVA followed by Tukey’s multiple comparisons test (*p* > 0.05)

*Abbreviations:*

*ESR* erythrocyte sedimentation rate; *RBC* red blood cell; *WBC* white blood cell; *ALP* alkaline phosphatase; *SGOT* serum glutamic oxaloacetic transaminase; *SGPT* serum glutamic pyruvic transaminase; *BP* blood pressure; *BUN* blood urea nitrogen

^a^ADAM, Inc.: http://www.nlm.nih.gov/medlineplus/encyclopedia.html (accessed October 2015)

^b^Mayo Foundation for Medical Education and Research: Mayo Clinic. www.mayoclinic.org (accessed October 2015)

A total of 45 AEs were reported during the 180-day study period: 9, placebo; 28, GC; and 8, UC-II (Table 10). The majority (62 %) of these occurred in the GC group. Fifteen of 45 events were classified as possibly related to supplementation, 14 of which belonged to the GC group and 1 to placebo. The 14 possible events linked to GC supplementation were primarily gastrointestinal in nature. The eight AEs noted for the UC-II cohort were deemed not related to supplementation. One individual in the GC group was removed from the study due to a respiratory tract infection (cough & fever). This infection was classified as an SAE. The event was investigated by the attending physician and center staff and judged as not related to GC consumption.

**Table 10:** Summary of analysis of adverse events in all subjects

	Study group		
	Placebo (*n* = 58)	GC (*n* = 65)	UCII (*n* = 63)
Severity			
Mild	7	21	5
Moderate	2	7	3
Severe	0	0	0
Relationship to Test Article			
Not related	8	14	8
Possible	1	13	0
Definite	0	1	0
Body System and AEs			
Gastrointestinal			
Acidity	2	3	2
Acute peptic disorder	1	0	1
Diarrhea	1	1	0
Epigastric burning	0	1	0
Febrile Enteritis	0	1	0
Heart burn	0	1	0
Vomiting	0	1	0
Nausea	0	1	0
Pain			
Arthralgia	0	1	0
Body pain	0	1	0
Low back pain	1	1	0
Neck Pain	0	1	1
Headache	2	4	0
Myalgia	0	1	0
Dermatology			
Itching	0	2	0
Xerotic skin	0	0	1
Pulmonary/Upper Respiratory			
Lower respiratory tract infection	0	0	2
Upper respiratory tract infection	0	1	0
Cough	0	2	0
Genitourinary			
Burning micturition	1	0	0
Burning sensation	0	0	1
Cardiovascular			
Palpitation	0	2	0
Constitutional Symptoms			
Fever	1	2	0
Insomnia	0	1	0
Total Number of Adverse Events Experienced During Study	9	28	8
Total Number of Subjects Experiencing Adverse Events: n (%)	7/58 (12 %)	20/65 (31 %)	8/63 (13 %)

## Discussion

We assessed the ability of UC-II to improve joint symptoms in moderate-to-severe knee OA subjects. The results presented herein demonstrate that individuals consuming UC-II presented with better clinical outcomes versus those supplemented with placebo or GC. Analysis of the WOMAC subscales showed that reductions in all three WOMAC subscales contributed to the improvement in the overall WOMAC score observed in subjects supplemented with UC-II. In contrast, GC supplementation failed to induce a statistically significant improvement in the WOMAC, VAS or LFI scores versus placebo. These results confirm previous findings by Crowley et al. [5], which reported greater reduction in knee OA symptoms after 90 days of UC-II supplementation than what was observed with GC.

An interesting finding that emerged from this study is that stratification, according to baseline COMP levels, appears to have selected for individuals that responded better to UC-II supplementation. A greater reduction in knee OA symptom scores was observed among individuals with baseline serum COMP levels ≥285 ng/mL and supplemented with UC-II. This improvement was of sufficient magnitude that statistically significant outcomes for UC-II were observed versus both placebo and GC supplementation under all the statistical analyses we employed (ANCOVA, MMRM and iAUC). COMP, a cartilage turnover marker, is released into serum by chondrocytes and synovial cells [10–12]. Levels of this biomarker have been shown in several studies to have modest correlation with OA severity. However, serum COMP levels in groups of OA subjects overlap with levels observed in healthy populations, and this has limited the use of COMP as a prognostic marker for OA progression [12–14]. While the biologic or clinical significance to these findings remains to be understood, we find this preliminary observation an interesting one suitable for further investigation and confirmation.

The etiology behind UC-II’s impact on OA symptoms is not known. However, undenatured type II collagen has been shown to protect animals against the onset of joint damage in both OA and RA experimentally induced arthritis models [15–18]. This protection is hypothesized to occur via the induction and migration of T regulatory cell (Tregs) to the area of inflammation and damage [19, 20]. The proposed role of Tregs may also have relevance to the moderation of OA symptoms, as *in vitro* studies have found that Tregs produce anti-inflammatory cytokines that stimulate chondrocytes to synthesize cartilage matrix components [21–23]. Additional studies that elucidate the precise mechanism through which UC-II mediates a reduction in knee OA symptoms are required.

The *in vivo* effects mentioned above may only be initiated by ingesting undenatured type II collagen as denatured (e.g., hydrolyzed) type II collagen fails to protect animals against the onset of arthritis [15]. This latter observation could explain why van Vijven and coworkers [24] concluded that there was insufficient evidence to support collagen for the treatment of OA as they combined data from all published clinical studies regardless whether native or denatured collagen was used in the trial.

We failed to observe any changes in knee ROM and distance walked regardless of supplementation. Improvements in these clinical outcomes are likely to be based not just on a symptomatic reduction in pain but also on physical improvements in the knee joint’s overall functionality. Until we undertake trials of longer duration, it remains an open question as to whether a slow acting supplement like UC-II can impact the biomechanical status of the OA knee sufficiently to improve knee ROM.

In the current study, GC supplementation did not significantly improve the signs and symptoms associated with knee OA. The scientific literature supporting the efficacy of GC is mixed, but there are various published studies which suggest that GC may moderate OA symptoms [25–27]. The GAIT study found that GC, and each component of GC individually, failed to impact OA symptoms as measured by the WOMAC pain scale; however, the placebo effect in that study was nearly 60 % which resulted in an underpowered study to determine differences between the treatments [28]. In contrast, a significant difference in knee pain was observed in the GC subgroup with moderate-to-severe knee pain compared to the placebo treated group [28]. To confirm the observation that GC may be more efficacious in subjects with moderate-to-severe knee OA pain, Hochberg and coworkers [29] performed a study in which OA subjects with moderate-to-severe knee pain, were randomized to GC or celecoxib for a period of 6 months. The results showed that GC treatment reduced WOMAC measured knee pain by 50 %, comparable to the results obtained with celecoxib [28]. It is worth noting that results such as these are not consistent across a number of studies for reasons yet to be determined [25–27].

In recent years, interest has focused on developing various biomarkers for monitoring OA progression and drug development [12, 30]. We therefore assessed several biomarkers of inflammation (CRP, IL-6 and MMP-3) plus cartilage breakdown (COMP) and found no significant change for any of these biomarkers in this clinical trial. Since OA appears to impact the biology of several key components of the knee (e.g., synoviocytes, chondrocytes, etc.), the ability to achieve a significant change in any one biomarker could prove challenging for a slow acting supplement like UC-II. Also, multiple factors including ethnicity, physical activity, gender differences, and diurnal variation influence these biomarkers resulting in large variability in their levels [31–35]. Therefore, any change in these markers would have to occur as a result of a highly significant impact on the underlying pathophysiology of OA, given that the correlation between these biomarkers and OA pathophysiology are weak [12]. Such effects might be expected to occur more readily with a targeted agent [4, 36].

## Conclusion

This study found that UC-II, a nutritional ingredient containing undenatured type II collagen, significantly improved knee function in OA subjects by day 180, compared to placebo and to GC, and was well-tolerated. Based on the data presented herein, we believe that additional research is warranted both to confirm and to define these findings more extensively.

## Abbreviations

AEs: adverse events
ANCOVA: analysis of covariance
ANOVA: analysis of variance
cGMP: current good manufacturing practice
CI: confidence interval
COMP: cartilage oligomeric matrix protein
CRF: case report form
CRP: C-reactive protein
GC: glucosamine hydrochloride plus chondroitin sulfate
iAUC: incremental area under the curve
IEC: Institutional Ethics Committee
IL-6: interleukin-6
ITT: intent-to-treat
K-L: Kellgren and Lawrence
LFI: Lequesne functional index
mITT: modified intent-to-treat
MMP-3: matrix metalloproteinase-3
MMRM: mixed model repeated measures
NSAIDs: nonsteroidal anti-inflammatory drugs
OA: osteoarthritis
PBO: placebo
ROM: range of motion
Tregs: T regulatory cell
UC-II: undenatured type II collagen
VAS: visual analog scale
WOMAC: Western Ontario McMaster Universities Osteoarthritis Index
